# Impact of a nurse-led intervention to improve screening for cardiovascular risk factors in people with severe mental illnesses. Phase-two cluster randomised feasibility trial of community mental health teams

**DOI:** 10.1186/1472-6963-10-61

**Published:** 2010-03-10

**Authors:** David PJ Osborn, Irwin Nazareth, Christine A Wright, Michael B King

**Affiliations:** 1 Department of Mental Health Sciences, (Royal Free Campus), University College London Medical School, Rowland Hill Street, London, NW3 2PF, UK; 2 Camden and Islington Mental Health and Social Care Trust, St Pancras Way, London, NW1 OPE, UK; 3 Department of Primary Care and Population Health, (Royal Free Campus), University College London Medical School, Rowland Hill Street, London, NW3 2PF, UK; 4 MRC General Practice Research Framework. Stephenson House, 158-160 North Gower Street London, NW1 2ND, UK

## Abstract

**Background:**

People with severe mental illnesses (SMI) are at increased risk of cardiovascular disease (CVD). Clinical guidelines recommend regular screening for CVD risk factors. We evaluated a nurse led intervention to improve screening rates across the primary-secondary care interface.

**Methods:**

Six community mental health teams (CMHTs) were randomised to receive either the nurse led intervention plus education pack (n = 3) or education pack only (n = 3). Intervention (6 months): The nurse promoted CVD screening in primary care and then in CMHTs. Patients who remained unscreened were offered screening by the nurse. After the intervention participants with SMI were recruited from each CMHT to collect outcome data. Main outcome: Numbers screened during the six months, confirmed in General Practice notes.

**Results:**

All six CMHTs approached agreed to randomisation. 121 people with SMI participated in outcome interviews during two waves of recruitment (intervention arm n = 59, control arm n = 62). Participants from both arms of the trial had similar demographic profiles and rates of previous CVD screening in the previous year, with less than 20% having been screened for each risk factor. After the trial, CVD screening had increased in both arms but participants from the intervention arm were significantly more likely to have received screening for blood pressure (96% vs 68%; adjusted Odds Ratio (OR) 13.6; 95% CI: 3.5-38.4), cholesterol (66.7% vs 26.9%, OR 6.1; 3.2-11.5), glucose (66.7% vs 36.5% OR 4.4; 2.7-7.1), BMI (92.5% vs 65.2% OR 6.5; 2.1-19.6), and smoking status (88.2% vs 57.8% OR 5.5; 3.2-9.5) and have a 10 year CVD risk score calculated (38.2% vs 10.9%) OR 5.2 1.8-15.3). Within the intervention arm approximately half the screening was performed in general practice and half by the trial nurse.

**Conclusions:**

The nurse-led intervention was superior, resulting in an absolute increase of approximately 30% more people with SMI receiving screening for each CVD risk factor. The feasibility of the trial was confirmed in terms of CMHT recruitment and the intervention, but the response rate for outcome collection was disappointing; possibly a result of the cluster design. The trial was not large or long enough to detect changes in risk factors.

**Trial Registration:**

International Standard Randomised Controlled Trial Registration Number (ISRCTRN) 58625025.

## Background

People with severe mental illnesses (SMI), such as schizophrenia and bipolar disorder, have elevated rates of cardiovascular disease (CVD) which is a greater cause of premature death than suicide [1-3]. Younger people with SMI are over three times more likely to die from a heart attack, while those between 50 and 75 years of age have a two-fold risk [3]. The reasons are manifold and include traditional cardiovascular risk factors such as smoking [4,5], diabetes [6] and dyslipidaemia [5,7,8]. Evidence regarding excess hypertension is more variable. Antipsychotic medications may exacerbate cardiovascular risk through glucose dysregulation, weight gain or dyslipidaemia [9,10]. People with SMI also eat less nutritious diets and are less likely to undertake even moderate levels of physical activity [4,11].

The physical health of people with SMI has become an international priority and in the United Kingdom (UK) both government and statutory organisations have emphasised the provision of physical health care and screening for people with SMI [12,13].

The UK National Institute for Health and Clinical Excellence (NICE) recommends routine screening for cardiovascular risk factors for people with SMI including both schizophrenia and bipolar disorder [14,15]. This risk assessment should be in line with recognised CVD risk prediction models such as the 10 year Framingham risk score [14]. Such models involve assessment of smoking and diabetic status as well as lipids and blood pressure. These individual risk factors are then combined with a person's age and sex in a CVD risk model which determines the overall likelihood of incident CVD in the next 10 years, as a percentage. The combined algorithms are more powerful predictors of future CVD than individual risk factors.

There is some debate regarding the best model of care for this screening and whether generalist or specialist models would be more effective. Generalist primary care services are more familiar with CVD screening, but may be less confident in working with SMI patients. Each general practice is only responsible for a relatively small number of SMI patients. Conversely, specialist Community Mental Health Teams (CMHTs) have expertise in SMI but are often unfamiliar with cardiovascular screening. The UK guidance recommends a model where this CVD care should be provided within primary care settings (general practice) but there is an expectation that specialist CMHTs and other secondary care psychiatric services will have a role in ensuring this screening has taken place.

There is a further expectation that people receiving antipsychotic medication should receive monitoring of their BMI and cardiovascular risk at start of treatment and periodically thereafter [9]. This might suggest a specialist secondary care model for screening. Conversely, in the UK general practices are given incentives to keep an SMI register and to provide patients with an annual review and health check [16,17]. However the contract does not specifically include screening for cardiovascular risk factors.

In England, secondary psychiatric care is organised according to the National Service Framework for Mental Health [18] and each geographical area is covered by one Community Mental Health Team, funded publically by the National Health Service. These CMHTs are multidisciplinary and each key worker has a caseload of people with SMI, whose care they coordinate according to the care programme approach (CPA). Regular review meetings are held under the CPA to review unmet needs and arrange appropriate biological, psychological and social management, including physical health. However since people with SMI receive both specialist and general practice services, there may be confusion about the provision of cardiovascular screening and other physical health initiatives unless communication is clear.

Evidence from clinical settings suggests that rates of CVD screening do not meet national SMI guidelines, either in primary or secondary care. A study of 1966 patients in UK assertive outreach teams revealed that only 26% had received screening for blood pressure, 17% for BMI, 28% for glucose and 22% for lipids [19]. In a different study in a primary care setting, people with schizophrenia were significantly less likely to receive screening for blood pressure or cholesterol as matched practice controls [20]. In the SMI group, 55.9% received a blood pressure reading in the preceding three years, 39.5% a weight and 12.3% a lipid measurement [20].

We developed a nurse-led intervention to improve rates of cardiovascular screening for people with SMI. Nurse-led interventions have proved successful in other areas of cardiovascular care, in both specialist and generalist settings. In the UK, a specialist nurse-led clinic for patients with diabetes demonstrated significant reductions in cholesterol and blood pressure in a randomised trial [21]. The intervention included regular 4-6 weekly reviews, including titration of drug therapies and lifestyle advice. An international literature review of nurse-led interventions concluded that general practice nurses have a potentially potent role in chronic disease management, including heart failure. However the authors stated that higher level evidence is required to determine and impact of such interventions and the most important roles and elements of such interventions [22].

We conducted a phase-two randomised feasibility trial to evaluate the short term impact of our nurse-led intervention. The nurse worked across the primary-secondary care interface to improve screening rates for people with SMI. In the UK, this collaboration between specialist CMHT services and general practice services is facilitated because each CMHT is aligned to a handful of defined general practices within the same geographical area. Primary and secondary care services share the care of people with SMI.

### Objectives and hypothesis

(a) To determine the short term impact of a nurse-led intervention to increase rates of cardiovascular screening for people with SMI in CMHTs, compared to 'treatment as usual' plus a CMHT staff education pack.

(b) To determine the feasibility of a full-scale randomised controlled trial of the nurse led CVD screening intervention in SMI.

We hypothesised that, compared to usual care, a CMHT-based nurse led intervention would increase the rate of screening for cardiovascular risk factors in people with SMI

## Methods

### Development of the Intervention

We undertook trial development work in line with the existing UK Medical Research Council framework for developing a complex intervention such as this [23]. Complex interventions are defined as those that include several components [23]. Our intervention included a number of elements including liaison with general and specialist clinicians, prompting them provide CVD screening (including smoking, blood pressure, random blood glucose and lipids), obtaining the CVD screening results and then providing screening where gaps or omissions were identified.

The development work for this intervention included interviews with service users, doctors, nurses and social workers from both primary and secondary care, all with the aim of designing an intervention to improve cardiovascular screening rates for people with SMI [24]. There was considerable variance in the preferences of professionals and services users regarding the best setting for the intervention and whether a primary care (generalist) model or specialist model should be provided. In qualitative interviews, most professionals believed primary care should take the lead, while service users were more divided, with a significant number preferring their mental health team to provide screening. No single model of care seemed to meet all professional and service user requirements, and a minority of mental health professionals were opposed to providing this form of care. The optimal model, addressing different these different preferences, was one which initially concentrated on primary care screening, with the back up of screening in secondary care when primary care screening failed to occur. Respondents in the development work stated that this "best model" would require dedicated coordination and extra capacity when CVD screening had not occurred in either primary or secondary care. When the option of an additional nurse to coordinate this work was discussed in the development work [24] most participants preferred this model.

On the basis of these findings we designed a nurse-led intervention based in secondary care but working across the interface between primary and secondary care. The intervention included three phases of screening. First, the nurse would promote screening in primary care by liaising with the general practice. Second the nurse would encourage CMHT workers to screen patients who had not received Primary Care screening after three months. Finally, if screening had still not occurred after a month, the nurse would offer screening to the patient herself.

### Study Design

This was a cluster randomised feasibility trial, with community mental health teams (CMHTs) as the unit of randomisation. We chose the cluster design for both clinical and methodological reasons. Clinically, the cluster design more closely mirrors real clinical practice. The methodological benefit of the cluster design is that it minimises the risk of "contamination" likely to occur in an individually randomised trial. This contamination would involve leakage of the intervention into the "Treatment as Usual" arm which might artificially elevate the rates of cardiovascular screening by General Practitioners (GPs) or CMHTs in the treatment as usual arm.

We compared two interventions to improve screening rates for CVD risk factors and the management of these risk factors among patients on CMHT caseloads. Teams in the intervention arm received the nurse-led screening programme as well as an education pack regarding appropriate screening for CVD related risk factors. Teams in the treatment as usual arm only received the education pack. We included the education pack to ensure that we were comparing the intervention with the best possible routine care.

### Setting and eligibility

We invited sequential CMHTs in Camden and Islington Mental Health and Social Care Trust in North London, to participate in the trial. Potential participating teams were selected at random from the existing 13 CMHTs in three geographical areas of the Trust, namely Islington, North and South Camden. We aimed to recruit two CMHTs from each area (total six CMHTs) which would agree to randomisation. The intervention was aimed at all patients whose care was coordinated by a key worker at an enhanced level on the general adult CMHT caseload. This includes those with a recorded diagnosis of schizophrenia, schizoaffective disorder, bipolar disorder, persistent delusional disorder, non-organic chronic psychosis or other severe mental illness in the clinical notes.

### Randomisation and allocation concealment

Participating CMHTs were randomly allocated to each arm of the trial using a sealed envelope method. A statistician uninvolved in the trial randomly generated treatment allocation numbers to which both the researcher and nurse were blind. The randomisation was stratified by the three geographical areas in the Trust, namely North Camden, South Camden and Islington. Once two CMHTs in each geographical area had agreed to participate, the envelopes were opened to determine the allocation for each CMHT. After randomisation it was not possible for the researcher, the CMHT workers or the patients to remain blind to allocation. The researcher could not remain blind to which arm the CMHTs had been allocated to when she assessed satisfaction with the intervention and who had performed screening (GP, CMHT worker or nurse). Therefore, during the evaluation phase, the possibility of observer bias was decreased by obtaining information on the main outcome directly from primary care clinical notes.

### Nurse-led intervention arm

The nurse was employed to work at the three CMHTs in the intervention arm. The nurse was a registered general nurse with previous experience of previous experience of providing cardiovascular screening. She did not have specific mental health qualifications.

The intervention lasted six months and targeted improving the levels of recording of the CVD risk factors required to estimate 10 year cardiovascular risk, namely smoking status, blood pressure and a non-fasting blood test for serum lipids and glucose. The main concepts of the intervention included establishing a system to monitor whether cardiovascular screening had occurred and sending prompts to primary and secondary care staff if screening had not occurred. Finally the nurse offered screening herself to cover patients who still had not received the complete battery of CVD screening. The elements of the intervention are contained in table 1. When the measurements were performed by the nurse herself they included laboratory tests for non-fasting serum levels of cholesterol, high density lipoprotein (HDL) cholesterol and random glucose. Current smoking status was recorded as well as resting blood pressure, measured by an automatic, calibrated sphygmomanometer. Body Mass Index is not part of the ten year cardiovascular risk scores, but it was calculated using calibrated scales for weight and standardised height measurement.

**Table 1:** Description of Nurse led intervention to promote screening for cardiovascular risk factors in people with SMI on the CMHT caseload.

Phase of screening	Components of intervention
Orientation	Write to all General Practitioner (GP) and community mental health teams (CMHTs) in the intervention arm to inform them of the nurse's work and the trial

	

Phase one: Liaison with primary care to prompt CVD screening Months 1-3	-Create secure lists and database of all patients under the care of CMHT, including address of GP. -Nurse writes to the GP of every patient explaining the rationale and evidence base for annual screening of cardiovascular risk in people with SMI
	i) Request that results of relevant CVD screening in the past year are sent to the nurse (including smoking and diabetic status, glucose, lipids, blood pressure and 10 year cardiovascular risk score). A proforma was provided.
	ii) Request that screening is arranged to assess any missing cardiovascular risk factors
	-When no response was received, up to two reminders were sent to the GP at 2 weekly intervals.

	

Phase two: Liaison with secondary care to prompt CVD screening Month 4	-Determine which patients have missing CVD risk factors on the nurse database. -Contact CMHT key worker for each of these patients. -Provide them with evidence and rationale for CVD screening in SMI -Request that they organise screening for missing CVD risk factors (including the same risk factors as phase one) -CMHT workers were provided with pathology request forms. -Request that they communicate the results back to nurse

	

Phase three: Invitation to cardiovascular screening with nurse Months 4-6	-Determine which patients have still not been screened by GP or CMHT key worker -Write to patient explaining importance of annual CVD screening -Invite patient to attend a scheduled CVD screening appointment with nurse at CMHT base. (including the same risk factors as phase one) -All results were sent to the patient and their GP

The nurse-led intervention did not include a theoretical behavioural change approach.

The teams also received an education pack which is described below.

### Comparison arm

CMHTs in the comparison arm did not receive any input by the nurse. However, they were informed of the nature of her work, the national recommendations for screening and which risk factors were required. They also received the education pack.

### Education pack

CMHTs in both arms of the trial were given an education pack including the following written information.

1) A summary of the evidence that CVD and CVD risk factors are more prevalent in SMI than in the general population.

2) Copies of guidelines and consensus papers which recommend the monitoring of physical health for clients with SMI

3) Information about CVD, appropriate screening for CVD risk factors and their abnormal thresholds and risk reduction interventions

4) A supply of patient booklets on CVD and risk reduction interventions

### Evaluation phase and Outcomes

Our primary outcome was the proportion of patients who had received screening for each risk factor by the end of the six month trial. The denominator was the number of people who had not been screened for each risk factor during the previous year. We also recorded who provided this screening and whether any interventions were received for identified risk factors. To decrease response bias in either trial arm, the main outcome data was defined as a record of screening in the primary care notes, rather than self-report. Trial process outcomes included the feasibility of i) recruiting and ii) randomising CMHTs to the trial and iii) obtaining outcome data regarding screening directly from patients and from their GP notes. The Secondary outcome was satisfaction with CVD screening, using the Client Satisfaction Questionnaire (CSQ-8) [25].

### Outcome data collection and sample size

The intervention lasted for six months and was offered to all patients on the CMHT caseload. Potential participants were contacted to obtain outcome data at the end of the trial. For the three months after its completion we collected outcome data on rates of screening from a sub-sample of patients in each of the 6 participating CMHTs. Potential participants with SMI were randomly selected from each CMHT caseload in batches of 20. The care-coordinator then confirmed whether the patient was eligible, according to the inclusion criteria below. Those eligible were sent a signed letter from the care-coordinator inviting them to attend a pre-arranged research interview. We aimed to recruit 75 participants in each arm of the trial within three months of the end of the nurse-led intervention. This number was chosen for exploration of trial feasibility rather than by a formal sample size calculation.

### Eligibility criteria for outcome interview

The inclusion criteria for the research interview were all patients with a diagnosis of SMI, aged between 18 and 75, on the CMHT caseload. We excluded those who were currently "too unwell" or unwilling to participate, which was defined i) by their care-coordinator or ii) by current inpatient admission or iii) receipt of home treatment from the crisis resolution team.

The majority of research interviews were conducted at the CMHT base and participants were reimbursed £10 for their time. Participants gave written informed consent to participate and permission was sought to access their GP notes and clinical record, to confirm whether screening had been performed and if any relevant interventions provided. An interpreter was offered if the patient did not understand English sufficiently.

### Measurements in evaluation interview

The structured research interview for consenting participants ascertained the following: 1) Demographic details, clinical diagnosis and current psychotropic medication, 2) Whether screening for each risk factor had occurred in the year before the trial and during the trial period 3) The date, content and provider of this screening by self report 4) Any CVD risk factors detected 5) Relevant interventions for each risk factor were recorded in participants whose BP, BMI, glucose or cholesterol were above normal ranges, or who were smokers. 6) The CSQ-8 [22]; which produces a score between 8 and 32; higher scores indicating greater satisfaction, and 7) Consent to check GP notes to confirm CVD risk factor screening, dates and any relevant interventions provided. After the interview, information about CVD screening and interventions was obtained from the participant's computerised GP notes, using a standardised form which was completed by the researcher (who visited the practice) or by the practice staff.

Data collection in the feasibility trial was time limited to three months after the intervention, due to funding of the feasibility work. This allowed two waves of recruitment of participants. The first recruitment wave occurred in all six participating CMHTs. However there was only sufficient time for the second recruitment wave to occur in four CMHTs (two in the intervention arm, two in the comparison arm).

### Ethical Approval

The trial was approved by the Camden and Islington Local Research Ethics Committee.

### Statistical methods

First, we compared demographic and clinical variables for participants from each trial arm and established how many had not been screened for each risk factor during the preceding year. For those who had not been screened, we compared the proportions with a GP record of each test by the end of the trial in each arm using logistic regression to generate odd ratios with robust standard errors to adjust for the cluster nature of the trial, age and gender. The unit of cluster was the CMHT. We also used Chi squared tests to compare the proportions with each risk factor who had received an appropriate intervention for it. Differences in satisfaction in the two trial arms were analysed by comparing mean CSQ scores using a t test.

## Results

**Figure 1:**
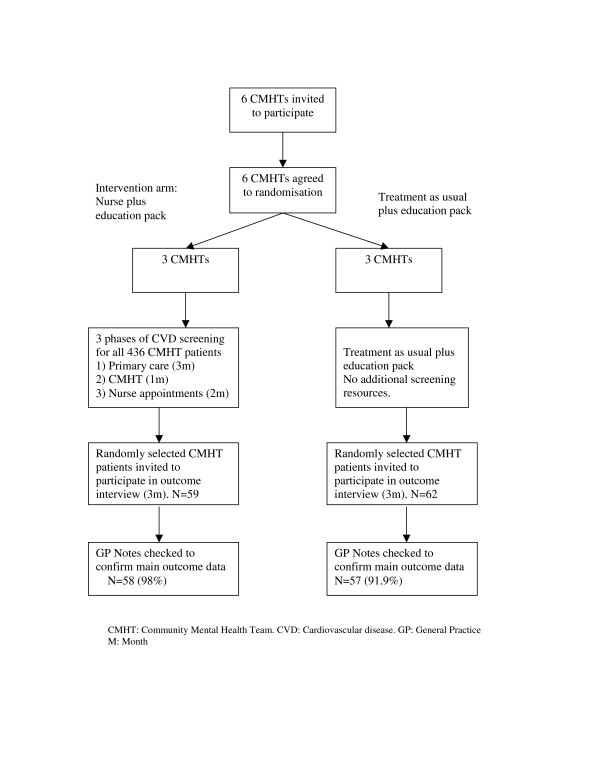
**Flow of CMHTs through feasibility trial**.

Each of the first six CMHTs approached agreed to participate in this feasibility trial. Figure 1 is an adapted CONSORT diagram for a cluster trial, showing the flow of CMHTs through the trial. The nurse attempted to organise cardiovascular screening for all of the 436 people in the three CMHTs which were randomised to the intervention arm.

### Response rates for research interviews

**Figure 2:**
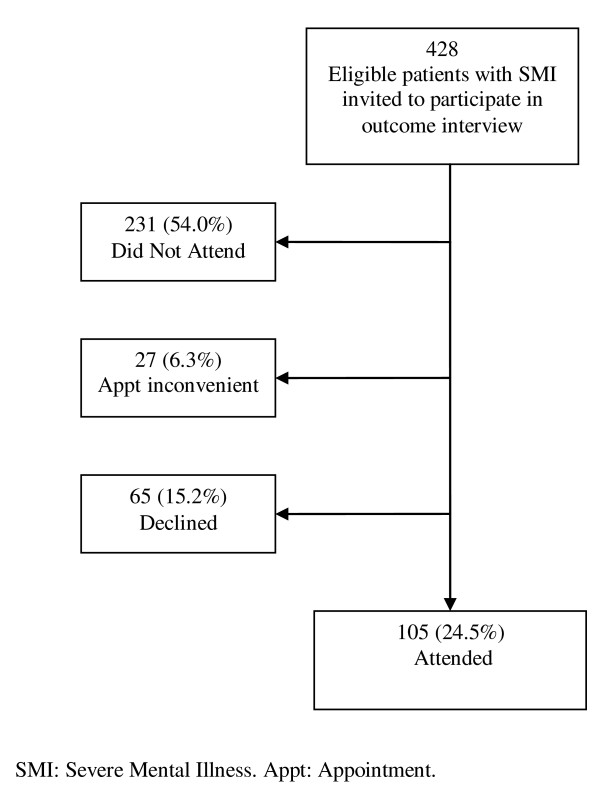
**First wave of recruitment to research interviews (Six teams involved)**.

**Figure 3:**
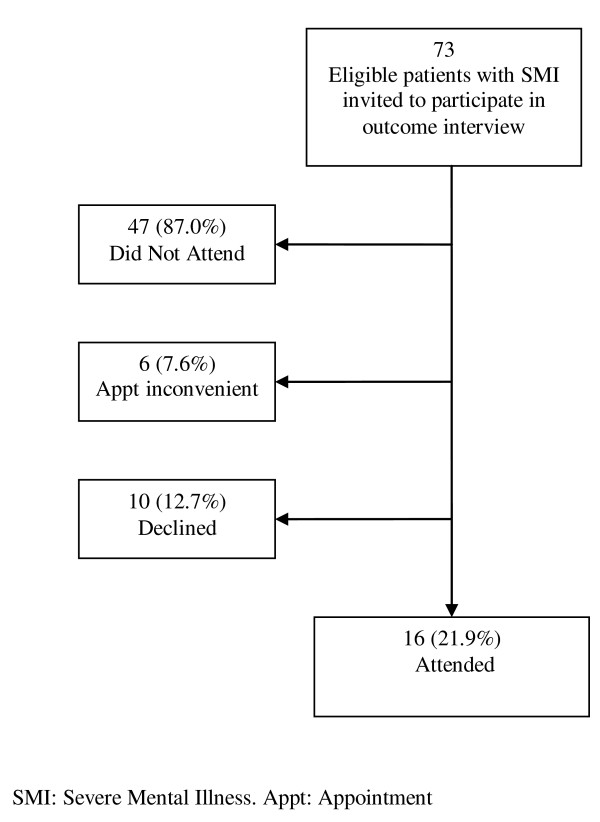
**Second Wave of recruitment to research interviews (Four teams involved)**.

A total of 121 research participants were recruited, 59 from CMHTs in the intervention arm and 62 from CMHTs in the comparison arm. The participation rates in the two sequential recruitment waves can be viewed in figures 2 and 3. 105/428 (24.5%) eligible participants attended interview in recruitment wave one (figure 2), and 258/428 participants were identified by care coordinators as eligible for re-invitation in wave two. In wave two, 73 of these eligible potential participants were invited in four CMHTs and 16 (21.9%) attended (figure 3).

### Comparison of trial arms

The demographic and clinical profiles of the 121 research participants from the two arms of the trial are compared in table 2. There were no statistically significant differences in the age, gender or ethnicity of participants in each trial arm. They had similar psychiatric diagnoses in their notes and reported no differences in current CVD risk factors. The proportions that had been screened for CVD risk factors in the year prior to the trial were also similar but low (results confirmed in GP notes). Less than a fifth of all participants had a record of BMI, blood pressure or smoking status in the previous year and about one in ten had received a blood test for glucose or lipids (table 2).

**Table 2:** Comparison of demographic characteristics of participants in each arm of the cluster trial

		Intervention Arm		Control Arm		Significance level (chi square except age = t test)
		(n = 59)		(n = 62)		
**Mean age (SD)**		42.0 (sd 10.52)		43.1 (sd 12.91)		0.600
Age range		21-65		20-67		

**Gender**						
Male		39	66%	34	55%	0.206
Female		20	34%	28	45%	

**Ethnic Origin**						
Asian		5	8.5%	2	3%	
Black		13	22%	9	15%	
White		37	63%	42	68%	0.243
Other		4	7%	9	15%	

**Employment Status**						
Employed		4	7%	3	5%	
Unemployed/looking for work		0	0%	1	2%	
Long-term sickness or disability allowance		43	73%	36	58%	
Retired		1	2%	1	2%	0.397
Looking after family or home		2	3%	1	2%	
Education		4	7%	8	13%	
Voluntary work		5	8%	12	20%	

**Diagnosis in clinical notes**						
Schizophrenia		29	50%	27	44%	
Bipolar Affective Disorder		13	22%	14	23%	0.291
Schizoaffective Disorder		4	7%	0	0%	
Other Psychotic Disorder		2	3%	4	7%	
Other diagnosis* (on enhanced level CPA)		10	17%	17	28%	

**Antipsychotics**						
First generation		11	19%	7	11%	0.323
Second generation		40	68%	38	61%	0.785

**Self-reported CVD risk factors**						
Current smoker		40	68%	39	63%	0.572
Overweight or obese		32	54%	37	60%	0.546
Raised cholesterol		18	31%	15	24%	0.436
Hypertension		10	17%	10	16%	0.903
Diabetes or raised glucose		8	14%	6	10%	0.505

**Screening received in year prior to trial**						
BP		6	10%	13	21%	0.103
BMI		5	9%	12	20%	0.850
Glucose		7	12%	5	8%	0.485
Cholesterol		7	12%	5	8%	0.503
Smoking status		6	10%	12	19%	0.167
10 year cardiovascular risk score		1	2%	2	3%	0.609

* includes depressive disorder, personality disorder or anxiety disorder.

SD standard deviation. BP. Blood pressure. BMI. Body Mass Index. CPA Care Plan Approach.

The rates of screening for each CVD risk factor during the trial are compared in table 3. Within the three months of the trial there were 6 patients (one in the intervention arm and 5 in the control arm) for whom it was not possible to arrange to review GP notes for practical reasons (n = 2) or lack of consent (n = 4). They were excluded from further analysis leaving 58 participants with confirmed outcome data in the intervention arm and 57 in the control arm (table 3).

**Table 3:** Proportions screened for each cardiovascular risk factor at six months.

CVD risk factor			Nurse arm (n = 58)		Control arm (n = 57)		OR	95% CI	**Adj OR**^**1**^	95% CI	Adjusted z test p value^1^
Blood pressure		N needing screen^2^	52		44						
		N screened %	50	96.2	20	68.2	11.7	3.5 - 38.4	13.6	3.9 - 47.3	0.001

Cholesterol		N needing screen	51		52						
		N screened %	34	66.7	14	26.9	5.4	3.2 - 9.1	6.1	3.2 -11.5	0.001

Glucose		N needing screen	51		52						
		N screened %	34	66.7	19	36.5	3.5	2.4 - 5.1	4.4	2.7 - 7.1	0.001

BMI		N needing screen	53		46						
		N screened %	49	92.5	30	65.2	6.5	2.0 - 21.6	6.5	2.1 - 19.6	0.001

Smoking status		N needing screen	51		45						
		N screened %	45	88.2	26	57.8	5.5	3.0 - 9.9	5.5	3.2 - 9.5	0.001

Framingham score		N needing screen	55		55						
		N screened %	21	38.2	6	10.9	5.0	1.5 - 16.5	5.2	1.8 - 15.3	0.003

Table 3 only includes participants whose GP notes could be examined to confirm screening.

1. Adjusted for age and gender.

2. Needing screening was defined as not having received screening for that risk factor within the previous year

CVD. Cardiovascular Disease. BMI Body Mass Index. CMHT Community Mental Health Team. OR Odds Ratio.

**Table 4:** Provider of screening in intervention arm

CVD risk factor	General practice		CMHT		Trial Nurse	
Blood pressure	24	48.0%	2	4.0%	24	48.0%

Cholesterol	17	50.0%	0		17	50.0%

Glucose	17	50.0%	0		17	50.0%

BMI	25	51.0%	1	2.0%	23	46.9%

Smoking status	21	47.7%	0		24	53.3%

Framingham score	5	23.8%	0		16	76.2%

By the end of the six month trial, the numbers who had received screening increased in both arms. In the control arm, around two thirds now had a record of BMI, smoking or blood pressure and approximately one in three had received a blood test for glucose and/or cholesterol (table 3). Participants in the intervention arm were significantly more likely to have received screening for all CVD risk factors during the trial, with an absolute increase of about 30% for each risk factor. Within the intervention arm, approximately half the screening had been provided by general practice and half by the nurse (table 4). No CVD screening was provided by CMHT workers during the month that they were asked to organise this. In the control arm, screening was nearly always provided by general practice (or infrequently by other medical services) with the exception of two patients for whom smoking status was recorded by the CMHT. There was no significant difference in satisfaction with CVD screening provision between the two arms (intervention arm mean (sd) CSQ-8 score: 24.7 (4.6), control arm: 23.4 (5.2); mean difference 1.5 (95% CI: -0.4-3.3) t test p = 0.12).

The study was designed to evaluate outcomes by recruiting participants at the end of the trial, rather than collecting data from CMHTs throughout the trial. However the cardiovascular nurse did report the proportion of the 436 CMHT patients in the intervention arm who required screening for each risk factor because they had not been screened in the previous year. She also recorded how many of these had been screened by the end of the six month intervention. These were as follows; 342 people had not received a blood pressure reading in the previous year and 259 (75.7%) of these were screened by the end of the trial. An annual cholesterol measurement was needed for 371 patients and 158 (42.5%) were finally screened. Random glucose was required for 342 and 189 (55.2%) were screened. Finally 350 people needed a BMI and 227 (64.8%) had been measured by the end of the trial.

Table 5 outlines interventions received by participants whose screening had identified CVD risk factors such as diabetes, smoking or dyslipidaemia. The majority had received lifestyle advice in both arms, and similar numbers were prescribed relevant pharmacological interventions and/or received further screening for the risk factor.

**Table 5:** Interventions provided during trial following detection of cardiovascular risk factors

		Intervention arm (n = 59)		Control arm (n = 57)		Chi square p value
High blood pressure^1^		(n = 10)		(n = 9)		
Re-measured		7	70%	7	78%	0.701
Diet advice		9	90%	9	100%	0.330
Exercise advice		8	80%	6	67%	0.510
Anti-hypertensive		7	70%	8	89%	0.313

Obesity^1^		(n = 32)		(n = 34)		
Re-measured		14	44%	19	44%	0.976
Diet advice		21	66%	23	68%	0.608
Exercise advice		21	66%	20	59%	0.569
Anti-hypertensive		6	19%	7	21%	0.851

Smoker^1^		(n = 39)		(n = 37)		
Advice		35	90%	32	87%	0.660
Referral to services		19	49%	18	49%	0.995
Nicotine replacement therapy		16	41%	19	51%	0.367

Dyslipidaemia^1^		(n = 18)		(n = 15)		
Re-measured		7	39%	5	33%	0.741
Diet advice		15	83%	12	80%	0.805
Exercise advice		10	56%	10	67%	0.515
Statin prescribed		7	39%	8	53%	0.670

Diabetes/raised glucose^1^		(n = 8)		(n = 6)		
Diet advice		7	88%	4	67%	0.347
Exercise advice		7	88%	2	33%	0.036
Oral Hypoglycaemic prescribed		5	63%	4	67%	0.872

1. Clinical diagnosis/status confirmed in General Practice clinical notes.

## Discussion and Conclusion

### Main findings

The nurse-led intervention, working to improve CVD risk factor screening in SMI across the primary-secondary care interface, significantly increased the proportion of CMHT patients who received such screening over six months. These results apply to the people who participated in the research interviews at the end of the trial. Rates of screening for all CVD risk factors increased for participants from both arms of the trial, but to a significantly greater degree in the nurse-led intervention arm. This resulted in approximately 30% more eligible patients being screened for each risk factor, including BMI, blood pressure and smoking status, as well as lipids, glucose and "Framingham" 10 year CVD risk score. However, even in the nurse-led intervention arm, a third of participants with SMI had not taken up a blood test by the end of the six month intervention and only a third had been apportioned a 10 year Framingham risk score. In the treatment as usual arm, two thirds had still not taken up a blood test and only one in ten had a ten year risk score. For those with identified risk factors, there was no difference in provision of relevant behavioural or pharmacological interventions between the two arms.

The baseline screening results highlight how few people with SMI currently receive CVD screening each year in general practice. Our findings were in line with previous research showing around one in ten people with SMI have been screened for cholesterol in primary care, in the last three years [20].

#### Strengths

We chose a cluster design, whereby the unit of randomisation was the CMHT rather than the individual, because this design prevents contamination in the treatment as usual arm. If patients had been individually randomised to the nurse, those who were randomised "out" might access better treatment than usual because CMHT workers would be aware that their patients were not receiving the care of the screening nurse.

The magnitudes of both the absolute and relative increases in screening rates were fairly consistent across all risk factors and all increases were of clinical significance.

#### Limitations

The response rate in the recruitment for outcome data was a major limitation of the study and this would need addressing in future trials. We aimed to recruit up to 75 people from each arm of the trial to assess the feasibility of outcome data collection, and fell slightly short of this target. The recruitment was time limited because of funding, but all SMI patients who were well enough received at least one invitation to a fixed research interview appointment in wave one. Recruitment of patients in CMHTs to research often requires intense effort and we did not have sufficient time for this. Consequently the participants who provided outcome data may have been a biased sample of CMHT patients and the generalisability of our results cannot be guaranteed. However the main findings rely on comparison of screening rates between participants from the two arms and there was no evidence of differential selection bias between participants from the two arms. They were similar in terms of demographics, diagnoses and antipsychotic medication. Most importantly there was no evidence that they differed in terms of their cardiovascular risk factors or their previous access to health care. Similar numbers of participants had received previous screening for CVD risk factors in both arms of the trial. Therefore there is little evidence of bias in the main risk factors for participants in each arm of the trial.

The intervention was designed to complement the configuration of United Kingdom primary and secondary care services and its international generalisability will therefore depend on local arrangements for service provision.

#### Feasibility findings

Although delivering the intervention proved feasible, a major burden in the trial was organisation and administration across the primary-secondary care interface. Although GPs often write to CMHTs, they do not regularly convey the results of physical health tests to CMHTs. The cardiovascular nurse spent many hours writing and requesting screening results from practices and whilst many were forthcoming, this was extremely time consuming. Some GPs also questioned the rationale for requesting this information and others felt uncomfortable being asked to perform physical health tests by a nurse from mental health services. This issue has partly been resolved by the latest UK NICE guidelines on schizophrenia [14] which state that CVD screening results should be passed between primary and secondary care in both directions. If the current intervention were implemented in CMHTs, administrative support would free up more time for the nurse to focus on clinical activity.

Six months was also too short a period for the nurse to be able to deliver interventions for people with elevated risk factors, since many of the screening results only became available towards the end of the period. Unless screening results can be obtained far more rapidly from primary care, future trials of this type of intervention would need at least a further six months to be able to demonstrate an impact on cardiovascular risk in people with SMI.

#### Clinical implications

This trial provides evidence that a nurse working in CMHTs has far more impact on rates of CVD screening than an education pack alone. Our findings also demonstrate the clinical need for such interventions; few participants with SMI had received annual screening for CVD risk factors prior to the trial. By the end of the trial, few patients in the control arm had received screening for all cardiovascular risk factors, despite the teams showing interest in the subject by participating in the trial and receiving the education pack regarding CVD screening. There was no evidence that CMHT workers provided CVD screening themselves in either arm of the trial. In the nurse-led intervention arm it is possible that staff were aware that screening would subsequently be provided by the nurse, so felt less compelled to organise screening themselves. However the lack of CMHT screening in the treatment as usual arm suggests that it may be difficult to achieve compliance with recent NICE guidelines which recommend that secondary care provides screening for any patients who have not received such care within general practice. Our findings suggest that this would require resource across the primary-secondary care interface and that basic training or education for teams would be minimally effective.

Improving CVD screening is only the first step in preventing cardiovascular disease and we need different trials to determine whether identified risk factors can be reduced by specialist or general interventions for people with SMI. Our trial was based in a specialist setting and it maybe that interventions set in generalist, primary care settings and run by practice nurses would be more successful in the future. This trial was not long enough for the nurse to provide interventions to effect behaviour change.

The satisfaction survey revealed high levels of satisfaction with physical health care services in both arms of the trial. This reinforces previous evidence that service aimed at improving the physical health of people with SMI are acceptable [26].

#### Research implications

The poor response rate for outcome data collection in our trial would need addressing in future studies and our study design would need major modifications. One option would be to return to an individually randomised design despite the potential for contamination of the intervention between the arms of the trial. If a cluster design were adopted, greater effort would be needed to permit outcome data collection in most SMI patients. This might require special ethical consideration. One possibility is that all SMI patients in a CMHT be asked for permission to record screening rates from their GP notes, perhaps with an opt out clause. However, any research requiring more complex outcome data collection, and interviews with patients, would encounter similar difficulties to the current study.

Future cardiovascular studies in SMI need to build on the brief intervention we tested. While the nurse-led intervention appears simple, it was organisationally complex. Challenges included working across the primary-secondary care interface and recruiting enough patients from CMHTs. Future trials need to be long enough for risk factors to be identified, addressed and then re-measured. The medium term end-point or outcome of such trials should be overall ten year cardiovascular risk scores. These trials need to be large enough for the proportions of people with identified CVD risk factors to generate statistical power to show differences in the reduction of ten year cardiovascular risk scores.

## List of Abbreviations

BMI: Body Mass Index; BP: Blood Pressure; CMHT: Community Mental Health Team; CSQ: Client Satisfaction Questionnaire; CVD: Cardiovascular Disease; GP: General Practice; MRC: Medical Research Council; NICE: National Institute for Health and Clinical Excellence; OR: Odds ratio; SMI: Severe Mental Illness.

## Competing interests

The authors declare that they have no competing interests.

## Authors' contributions

DO IN and MK designed and led the study and were the grant holders.

CW collected the outcome data, undertook the development work and helped design the intervention and manual, supervised by DO IN and MK. DO and CW analysed the data with support from IN and MK. All authors contributed to writing the final manuscript and all have approved it for submission.

## Pre-publication history

The pre-publication history for this paper can be accessed here:

http://www.biomedcentral.com/1472-6963/10/61/prepub
